# A Different Conversation: Psychological Research and the Problem of Self in Autism

**DOI:** 10.1007/s12124-019-09506-2

**Published:** 2019-11-23

**Authors:** Alessandra Fasulo

**Affiliations:** Department of Psychology, University of Portsmouth, Portsmouth, UK

**Keywords:** Autism, Narrative, Self, Jerome Bruner, Conversation analysis, Paradox of autistic self

## Abstract

Observations about peculiarities in the autistic population concerning type and frequency of references to subjective states, and lack of perspective taking, have been on the whole referred to as the paradox of the autistic self, i.e. a co-presence of ego-centeredness and weak self-referentiality (Lombardo & Baron Cohen 2010). Prevalent approaches in autism ascribe these peculiarities to high order disfunctions caused by neurological factors, such as defective self-encoding processes. Two narratives told by an adult man with Asperger during counselling are examined with Conversation Analysis; the analysis identifies features that may lead to descriptions like the paradox of autistic self, but also reveals competences related to perspective-taking and narrative construction. Drawing on Bruner’s narrative theory, as well on recent interactional research on autism and the psychology of self, it is suggested that a relatively limited practice with narrative co-construction might be at the origin of the peculiarities observed. A socio-developmental approach to the understanding of autism not only can provide explanations compatible with first and second person accounts of life with autism, but can also open new paths for researching ways of self-construction that are less reliant on social interaction. The article finally challenges assumptions in psychological research about the ability of humans to access their internal states, and discusses how such assumptions can deter understanding of atypical populations.

## Introduction

The expression ‘paradox of the autistic self’ (Lombardo and Baron Cohen 2010) was coined to summarise findings from experimental studies according to which people with autism are at the same time egocentric, i.e. predominantly self-interested and unable to consider the perspective of others, and with a weak self-referentiality, i.e. with scarce ability to access and express their own cognitions and emotions. A large proportion of those studies, as common for autism research, were conducted with children or young people as participants, because autism tends to be considered a developmental disorder, but ego-centredness and weak self-referentiality - as well as difficulties with narrative discourse – have been associated to autism without specification of age. These descriptions, however, are hard to reconcile with other sources of evidence, for example the abundance and quality of autobiographical material produced by autistic authors^1^: for people supposedly uninterested in others, who have difficulties with narrating and low access to their interiority, this is no obvious endeavour.

In this article I will discuss two story-telling episodes narrated during counselling by a middle-aged man with Asperger, using Conversation Analysis. In examining these conversational fragments, I will attempt to illustrate how perceptions of ego-centredness and weak self-referentiality may arise in observers or co-participants, but also that different levels of interpersonal coordination and expressions of subjectivity can be identified. Jerome Bruner’s work, to which this special issue is dedicated, will guide the exploration of the problem of the self in autism.

### Perspectives on the Autistic Self

Observations related to the paradox of the autistic self, include, for the egocentric part, autistic people occupying long stretches of conversation with topics of their own interest, not inquiring about the other person, and being unable to read and react appropriately to others’ emotions, intentions and motives. Such observations are globally also referred to as an impairment in ‘Theory of Mind’ (Baron-Cohen 1997). For the ‘weak self’ part of the paradox, sometimes also called ‘absent self’ (Frith 2003), the key observations concern difficulties with the 1st person pronoun ‘I’, infrequent mention of emotional or cognitive internal states; low introspection, (Gillespie-Smith et al. 2018) and limited episodic autobiographical memory (Brown et al. 2012). Evidence for both ego-centredness and weak self-referentiality often comes from narrative tasks, in which people with autism, compared to control groups, are found on the one hand less likely to include information that would help listeners, such as temporal references, thus indicating less perspective-taking work, and, on the other, to associate less complex and varied mental and emotional states to both their own or other characters’ actions (see Stirling et al. 2014 for a review). Lombardo and Baron-Cohen (2010), reviewing the kind of evidence reported above as well as neurological observations, hypothesise that both parts of the paradox could be explain by a single deficit in the ‘neural circuitry coding for self-representations’; this would hamper the development of the self-other distinction as well as self-awareness and the theory of others’ minds.

Apart from criticism about the adequacy of an all-explanatory, single core deficit in autism (Roth and Rezaie 2011, cit. in Scholiers 2019), experimental findings are contradictory when looking at either narrative abilities (Colle et al. 2008) or other types of self-processing tasks (Gillespie-Smith et al. 2018), with definitive results about systematic poorer performances in any of those areas not having been ascertained. Secondly, some of the difficulties in communication and social relations reported in children and youth have been often observed to fade or disappear with age or intervention (Vivanti et al. 2018), which is at odds with theories of a neurologically-based impairment in those areas. Finally, autobiographical accounts of people with autism and research in interactional settings (see Fasulo and Sterponi 2016, for a review) offer evidence difficult to reconcile with the descriptions reported above.

An example of a personal account challenging the idea of the weak or absent self can be found in Jim Sinclair’s words directly addressing this issue:

‘I have an *interface* problem, not a core processing problem. I can't always keep track of what's happening outside myself, but I'm never out of touch with my core. Even at worst, when I can't focus and I can't find my body and I can't connect to space or time, I still have my own self. That's how I survive and how I keep growing.’ (Sinclair 1992)

The sense of his own self is for Sinclair phenomenologically very strong, so much that he feels anchored by it when connections to the outside world become insecure. This example suggests that perceptions of a weak or absent self may have to do with the the expectations on, and interpretation of, social and communicative behaviour in people with autism from the non autistic social environment. Dawn Prince (2013:329), for example, reports how the strive to adapt to the neurotypical world can suppress spontaneity and the opportunity of rich social relations:

‘The ways we pass as normal keep us from having any of our three deepest wishes granted like heaven; we can’t be loved for who we are, because we hide ourselves, knowing we are freaks; we can’t give, because we are often too afraid; and because no one knows who we are or what we can give, we are afraid to die, knowing we can’t truly be remembered. […]. It is from this unnatural hiding, hoarding, our hairless cowering, that all regrets and shame flow.’

Prince, who is an autistic anthropologist and primatologist, has also offered striking accounts of autistic diversity as such (Prince-Hughes 2004), but the words above reveal that personal annihilation came for her from the fear and lack of recognition that characterized her social exchanges.

The inadequacy of theories about neurologically weak or absent selves is also flagged from within neurobiology:

‘self-referential coding is the foundation of human consciousness. […] Impairment in self-referential coding will also mean that autists [sic] are barely conscious and living in peaceful state of diffused consciousness (pain is based on a sense of self, locality). It is most likely the exact opposite of autists. They are in an extremely localized state, extremely aware of themselves, extremely aware of others and in a battle for their life to hold back the intensity and pain of it all’ (Markram and Markram 2012,2)

Markram and Markram's description is consistent with personal accounts and conversations with people of autism, who, as we have seen, commonly report an acutely felt presence of others and simultaneously a preoccupation with one's self conduct and presentation (see for example the interviews in Biklen and Attfield 2005). In the following I will be re-examining concepts related to the paradox of the autistic self using a Conversation Analysis approach to analyse data of interaction during counselling sessions of a person with autism.

## Method

### Method of Analysis

Conversation Analysis (CA) is a discipline born in sociology for the understanding of social interaction (Sacks et al. 1974), and is now broadly multidisciplinary (Sidnell and Stivers 2012). One of its strengths is that it invites suspending assumptions about how individuals would act on the basis of their pre-assigned labels such as age, gender or diagnostic categories, to focus instead on how interactants produce their turns at talk to manage the job at hand, and how they display understanding of previous turns from others in their subsequent verbal or non verbal acts. In this way, Conversation Analysis can access what is the current ‘context’ for participants as they co-orient to it, and thereby create it, with each successive communicative move.

CA has uncovered the principles of ordinary conversation and demonstrated the robustness of its findings across different languages and settings, and is therefore uniquely positioned to evaluate the conformity and efficiency of interactions that might be in some way ‘atypical’ (Antaki and Wilkinson 2012).

### Sources of Data

The two conversational fragments under examination were extracted from two different counselling sessions in which the client is a man with Asperger in his early fifties. The client, whom we shall call Francis, is unemployed, although he participates as a volunteer to autism advocacy and political associations, as well as being an active research participant and collaborator. He and the counsellor met through a mental health charity organization; they are of similar age and both British. The counsellor, here called Darren, had worked with autism in a younger population, and offered Francis a number of free sessions in exchange for experience with autism in adults.

Counselling is a natural environment for discursive explorations of the self. Furthermore, it is a type of ‘institutional talk’ (Drew and Heritage 1992) that does not entail a rigid turn allocation system, thus allowing also to analyse the sequential coordination of speakers across turns at talk.

The sessions had been filmed by the counsellor for his own and the client’s record, for a duration of 18 months. The entire set of recordings was donated for decision of the client to the Autism Research Network of the University of Portsmouth. Informed consent for the study have been obtained from both.^3^ The first and last 10–12 min of 20 sessions, evenly distributed across the 53 total sessions have been transcribed according to the Conversation Analysis notation system (Jefferson 2004; see Appendix). The two extracts that will be used in this article are from the initial segment of the 17th and the 3rd session respectively. All names and references that may lead to the recognition of the identity of the participants have been changed.

### The Shallow Self-Narrative

Extract 1, analysed in this section, is an example of what we may call a ‘shallow self-narrative’, whichmay be perceived as demontrating low self-referentiality. It starts 7 min into the meeting, and the client is reporting on a phone call with a newly met relative.

The only additional symbol to the Jeffersonian transcription notation is the bracket in italics used to indicate overlap between verbal and non-verbal actions. If there is no overlap sign it is intended that the non-verbal action occurred sequentially after the last transcribed verbal part.

Extract 1)

[Session 17 INI [07:27:00] FR: Francis, client. DA: Darren, counsellor]

1. FR: ((Sits back on his chair, eyes closed, head reclined back))

2. I had a curious phone call last (.) Wednesday- evening.

3. ((raises head))

4. DA: Mhmh.

5. FR Anduh:, (0.2) it was this chap who was a relat= I don’t-

6. I don’t know if I told you I had to miss a session

7. at the beginning of November because I had to go

8. to a funeral down in Bristol. ((opens eyes looks at DA))

9. DA: Yeah.

10. FR: Uhm well this chara=person who di:ed uhm, his-

11. some relative of his who sort of dealt with the matter

12. rang me last Friday.=Wednesday night,

13. out of the blue:, saying that he wanted my::-

14. to [confirm my addre:ss, >↑which ↓I did.<

15. [((*raise arm, points sideways*))

16. because he said there’s a little check coming fo:r me:.

17. (0.6)

18. it’s only five pound it’s js five pound°

19. (>by this character’s estate<) so I said

20. well >thank you very much<

21. and there was also a curious invitation. (0.2)

22. from this chap ((pinches the top of his nose, hand covers the face))

23. he a:sked, (0.5) if I’d been in touch with various uh:

24. (I suppose) actually

25. (1.8) ((rotates extended hand as indicating ‘more’))

26. (relatives of his) one in Cambridge, uhm

27. and one in ↑O::xford.

28. DA: Mh.

29. FR: And I said well quite frankly I haven’t had time

30. DA: Mh.

31. FR: And he said well he wants me to go and see them

32. when in Cambridge and in Oxford so I’ll do tha:t.

33. DA: Mhmh.

34. FR: And uhm,

35. (1.0)

36. So that was quite go:od,

37. DA: So what do you think about these relatives

38. you haven’t seen for a whi:le.

39. FR: °I didn’t even know they existed >to tell you the truth°<=

40. =but they seemed quite pleased to- keen to m-

41. continue to make contact with me so I suppose

42. I’m gonna do=it.

43. DA: Yeah.

44. FR: Uhm a:nd, ((covers his face with the hand as before,

45. pinching the top of his nose, head down))

46. DA: That’s=that’s nice [( )]

47. FR: [I was- yes, I was supposed also

48. this- I didn’t tell you, ( ) this kitchen business

49. ((continues with the kitchen topic))

In reading this account, we might have some trouble, just like the counsellor, in getting a sense of the personal meaning that the contact with this relative had for the client. In introducing the event, Francis qualifies it as ‘curious’, a term that refers to the unexpected or slightly puzzling nature of the event, but without a clear positive or negative slant. He then reconstructs the episode by replaying the phone dialogue in detail, and reports his intention to comply with the invitation to meet the other relatives in Oxford and Cambridge. He ends with the evaluative phrase ‘that was quite good’, which is not framed in personal terms (e.g. the way ‘I was pleased’ would be). The counsellor (who knows that Francis has lost almost all his family members, so discovering a new relative might have been noteworthy), solicits at this point Francis’ personal take on the event (lines 38–39); Francis makes light of it, replying that he did not know of the relatives’ existence, but since they are ‘keen’ to meet he ‘supposes’ he is going to do so (lines 40–43). The counsellor then offers himself a positive characterization: ‘That’s nice’ (line 47). The continuation of the counsellor turn is made unhearable by Francis’s introducing a different topic in overlap, and covering his face in a gesture of concentration. An impressions of weak self-referentiality, such as those reported in experimental observations, could thus derive from the way this narrative maintains a factual narrative plane, devoid of explicit references to internal states.

The sequential analysis of the extract, however, shows also a series of elements documenting both personal stance and perspective-taking. As far as perspective-taking is concerned, we can see that Francis takes into account the knowledge of the counsellor in setting up the story, and includes discursive devices that prepare him to the fact a narrative is coming, help him tune in to its genre and anticipate the point: first, there is a ‘story preface’ (Sacks 1992) that introduces the story and its gist: ‘I had a curious phone call last (.) Wednesday- evening.’ at the end of which Francis raises his head and waits for the counsellor ‘go ahead’ (‘Mhmh’). Then Francis starts introducing the caller, but takes a step back in order to place the event within the counsellor’s time coordinates (i.e. the missed therapy session, lines 5–8); he also abides to principles of economy in person identification, using a level description suited to the counsellor’s knowledge (line 11, Sacks and Schegloff 1979). The point of the story, i.e. the invitation by the relative to meet up with other members of the family, is marked by the repetition of the assessment ‘curious’ (line 2): evaluative marking of key points support the recipient’s interpretive work and help them react at appropriate times, while also displaying ‘tellability’, namely what makes the story worth telling; finally, a canonical general evaluation rounds up the narrative and exits the story world (Labov and Waletzky 1967; Sacks 1992).

A misalignment in intersubjectivity seems rather to have occurred at the sequential juncture where the narrative ended. Here the counsellor’s bid to expand on the personal meaning of the encounter was not taken up, and his assessment of the event (‘That’s nice’) was not acknowledged or seconded (Pomerantz 1984), apart from the quick ‘yes’ embedded in the turn that initiates the next topic. This turn covered the counsellor’s continuation, and, together with the postural and gaze withdrawal, prevented any further comments on the phone call story, curtailing the therapists’ opportunities for using the story as counselling material.

Thus, on the one hand, the better part of the work that makes for an interactionally efficient story-telling was performed, but, on the other hand, Francis stopped short of including elements of the ‘internal landscape’ (Bruner 1986), either spontaneously or as a response to the counsellor’s elicitation. The first consideration is that procedural intersubjectivity and expression of internal states should be kept analytically and theoretically separate, but we can perhaps also go a step further and distinguish between the way personal relevance is embedded in the story and the way a story opens up to external interpretations.

In terms of how the story was initially introduced, it is worth considering that it was told at the beginning of the counselling session, when it is customary for this dyad to focus on the week just ended; Francis would report episodes relevant to the shared counselling agenda, especially in terms of how he dealt with his difficulties, many of which involve coping with practical commitments and social encounters. In light of this, the story about the telephone conversation can be seen as a report on how Francis managed successfully a phone call arrived ‘out of the blue’ from a person he had only briefly met once. Francis described how he dealt with the situation both pragmatically and socially, confirming his address (a passage emphasised with gestures), and agreeing with the prospect of future meetings with yet more unknown relatives.

The prospect of a social occasion with strangers is not necessarily or completely positive for a person with Asperger: it almost certainly would involve the stress of travelling and its preparation, as well as navigating the social event itself. At the same time, there are indications that neither the relatives, nor the person who died retained a great personal significance for Francis: the person who died was initially referred to as a ‘character’ then corrected as ‘person’, and the relative is consistently called ‘this chap’. So, if we stay with what *is* in the story rather than in what is *missing* from it, we may see that for Francis the tellability of this story has more to do with the very fact of the conversation happening the way it did, and the good will he demonstrated by agreeing with the proposed plans, rather than in the emotional resonances of the contact with the relatives: ‘That was quite good’: *that* – dealing with a ‘curious’ social event - went well. Francis is not going to *make more of it* than was actually there for him. So in terms of elements of personal relevance that can be heard in the story, there could be a problem of discrepancy between what may be noteworthy, or ‘exceptional’ (Bruner 1991; see also Smorti 2019) enough to deserve being storified, for people with autism and those without the condition. Autism research is starting now to explore the limitations in neurotypicals’ understanding of the autistic mind, and this could be an example of the way in which the personal significance of mundane events is not part of the shared tacit knowledge between people with autism and people without. This, together for the proclivity to embody stance and motives within characters’ action description rather than with explicit psychological terms (Bottema-Beutel et al. 2017) might account for the perception of the ‘shallow narrative’ in an interlocutor.

If differences in shaping and interpreting stories may induce false interpretations of lack of self awareness, the relational disconnectedness evidenced in the foreclosure of the narrative to the interactant’s contribution is of a different nature: it is a barrier halting the merging of subjective perspectives, hampering the possibility to resolve discrepancies in the way a story is interpreted. The narrative was not offered as a bid for joint meaning-making but as a self-enclosed unit, comprised of its definitive meaning After the analysis of the second extract we will come back this observation in light of Jerome Bruner’s theories about narrative and the self.

### The Exorbitant Narrative

The second extract starts early in the 3rd session. The counsellor is proposing to discuss the client’s relationship with his former psychotherapist, but stops mid-sentence to ask Francis how he should call her throughout. This triggers an interlocked series of short narratives, after which the counsellor resumes his talk.

The background of this conversation is that Francis, after a severe breakdown during his undergraduate study, in the 80s, was offered by the National Health Service a psychoanalytic psychotherapy. The psychotherapist was a woman doctor (here renamed Margie Burrow). After about 1 year, the therapy was interrupted because of a change in Dr. Burrow’s assignment. Francis believes that his psychological well-being had been improving substantially up to that point, thanks to the strong transfer he had developed for her, but that the interruption had caused a major set-back. All his subsequent efforts to continue the therapy or get a similar one with a different doctor were unsuccessful, hence the strong resentment with the medical authorities that seeps through the second part of the sequence (Extract 2b).

Extract 2a [Session 3 INI 2:47–3.50]

01 DA: Okay so what we’r:e gonna j’z (.) talk about

02 is really y-, (0.3) your relationship with-

03 (0.8) would you call her Burrow or Ma:rgie=

04 how would you refer [to her

05 FR: [Well

06 *[*((*puts hand over nose bridge, covering his eyes*))

07 *[*I=I’ll tell you uhm when it started off

08 I didn’t refer to her as anyth:i::ng,

09 DA: [(and tha-)

10 FR: [then it was Doctor Burrow and then=

11 =well- I >said to her in one session towards the end

12 that I’m< (.) when I tried to- you know

13 >I’d write in the diary< *[*the next appointment

14 *[*((*looks up at D*A, *rotates hand,*))

15 [>for exam-<

16 DA: [°Yeah.

17 FR: *[*First of all I’d write uhm Doctor *[*Bu:rrow::,

18 *[*((*head back on the chair, gaze up [right thumb out in listing gesture*))

19 Then it was Doctor *[*Ma:rgie Bu:[rrow:,]

20 *[*((*right index finger out*))

21 DA: [°Yeah.]

22 FR: *[*>Getting more and more a:ngry,<

23 *[*((*smiles, left hand raises up enacting the doctor*))

24 DA: Yeah.

25 FR: Then it was Margie Burrow- *[*>↑white<

26 *[*((*raises open hand*))

27 =then was Margie, can you see? >white face< ((*raises hand*))

28 that was an *[*indication of my fee:lings >developing<

29 *[*((*turns head toward Darren and looks at him*))

29 and trying [to realise] at the time=so.

30 DA: [Yeah.]

31 FR: Uhm >sorry< uhm >yeah.<

32 DA: [Tha:t’s fine,

33 FR: [Call her Doctor Burrow, whatever you like.

The counsellor’s question about the Doctor’s name is a ‘utilitarian’ one, within a turn that is hearably a prelude to a longer spate of talk; the question, however, turns out not to have a simple answer for Francis. He recalls how he changed the way he called the Doctor (within himself) throughout the therapy, progressively using more intimate names; this was visible to the Doctor when Francis wrote down in the diary the date of the next appointment, and he recalls how it made her angry (see the ‘white face’ and hand raised to enact the therapist, lines 23–27^4^). The counsellor’s ‘Yeahs’ (lines 16, 21 and 24) are an indication that he is treating this as a soon-to be-concluded topic, after which he will take the floor again (Jefferson 1984). At the end of the extract Francis indeed apologises, seemingly for the overabundant interlude. The apologies are accepted (‘No, that’s fine’), and Francis provides a conclusive answer to the original question ‘Call her Doctor Burrow, whatever you like.’ Additional recollections, however, starts to be narrated at this juncture, concerning the respect attached to the Doctor’s name by other medics. A series of caustic remarks follow, played as a dialogue between Francis and the medics, around how the latter were not able to help him in his predicament of having lost the psychotherapist, and how this caused him later being sectioned in a psychiatric hospital.

Extract 2b

33 FR: BUT- tell you wha- her (0.2) bosses were always so official=

35 =it was Margie this Margie tha:t, [Margie

36 DA: [Yeah.

37 FR: the <greatest psychothe:rapist since Freu:d,>

38 so I said well I should see her then=

39 =no answer to tha:t.

40 DA: Yeah.

41 FR: And=uh=and Freud >actually didn’t run out

42 on his patients and put them in- where they put me:<

43 (.) except of course he was thrown out of Austria in

44 nineteen[thirtyeight hahaha

45 DA: [Yeah uh=hm

46 FR: They didn’t like that either he[hh

47 DA: [Ye:s.

48 FR: Sorry hmhm? ((*leans towards counsellor*))

49 DA: No th=tha[t’s-

50 FR: [( )

51 DA: =What we’re trying to do is [work on that relationship

55 FR: [°Yes:°

The narratives in this second part proceed incrementally, and are inflected with witticism. Francis begins initially associating another memory to the topic of the Doctor’s name, and replaying fictionalised snippets of his conversations with the medical authorities after Burrow’s departure. Then the comparison between the Doctor and Freud gives him the chance to remark on the abandonment and the consequences he suffered from it, and this finally drives a more disjointed comment about Freud’s own vicissitudes, still performed as part of the dialogue with the medics, including their hostile responses.

At the junction after each part, the counsellor utters again his Yeah’s (lines 36, 40, 45, 47), exerting some pressure toward resuming the topic he had initiated earlier. Similarly to the end of the naming narrative in Extract 2a, this spate of talk ends with an apology from Francis, and a more definite handover of the floor marked also by the leaning posture (although Francis utters some more words in overlap with the counsellor’s, so we do not yet see a completely neat closure). The therapist then continues on with the initial project, which is restarted by using the same words of the beginning (line 51).

The ‘naming’ episode taken as the whole (Extracts 2a and 2b) shows almost opposite features to the story in Extract 1 about the newly met relative. There, the narrative seemed to offer insufficient personal meaning; here, there seems to be an overabundance of meaning that makes the talk exorbitant, causing a long detour from the conversational trajectory set out by the counsellor at the beginning of the extract. Francis’ response to the question about how to call the Doctor went straight into the traumatic experience of more than 25 years earlier, as he mentioned his growing attachment to the therapist, the brusque separation, his frustrated attempts to persuade the medical authorities to let him see her again, and his hospitalisation (‘where they put me in’). Emphasising the respect of ‘her bosses’ towards the psychoanalyst also adds weight to the loss Francis endured, and is evidence in favour of his claim that the therapy had been beneficial. These contents are all relevant to the topic the counsellor had attempted to initiate, i.e. Francis’ relationship to Doctor Burrow, and demonstrate the ability to identify feelings behind one’s conduct.

In terms of perspective-taking, again we have two sets of observations. On the one hand, those stories were displaced in a way that might look egocentric vis-à-vis the therapist conversational project: they were appended to the naming question in the middle of the introductory turn of the counsellor and created some awkwardness, with the therapist stalled and proffering ‘yeahs’ that are typically used in to curtail a speaker’s production, and Francis’ repeated apologies. In any case, because of the sequential position, those contents did not become available for counselling. On the other hand, Francis’ apologies themselves display awareness of the oversized response, and the packaging of the narratives as asides (i.e. concise, fast delivered self-contained anecdotes) are consistent with their sequential location. Other indicators of collaborative (and competent) speakership can be observed in the conversational devices that prepare the listeners to the response going somewhat off track, like the turn beginning ‘Well’ (Extract 2a line 5: Heritage 2015) and the ‘I’ll tell you’ preface (line 6: Schegloff 1992). The closing line finally responding to the specific question about how the counsellor should call the doctor (line 33) complies with the initial request of the counsellor, and the emphatic handovers of the floor displays recognition of the counsellor’s speaking rights. Similarly to Extract 1, then, intersubjectivity at the procedural level – the moment-to-moment attunement about what is going on right now (Schegloff 1992) – is present throughout, but the timing and pacing of the narratives did not incorporate entry points for the interlocutor, or an orientation to the contents expressed having been disclosed for comment and interpretation from the other. This is different not only from typical psychotherapeutic interactions, in which there is an expectation that the therapist interprets what the client has related, but also from ordinary conversational story-telling, in which listeners’ contributions are almost always present towards the ending (Sacks 1992), and often also at various points throughout (Lerner 1992), especially after evaluative segments (Goodwin 1986; Mandelbaum 2003). Francis’ stories instead, again, were delivered as finished products and not as a framework for joint problem solving (Ochs et al. 1989).

Once the orientation to the other in narrative talk is lifted, other motives can overtake, like emotional relevance, or care for precision: for example, the counsellor in Extract 2a has asked ‘would *you* call her Burrow or Margie how would *you* refer [to her’(emphasis added), so Francis explained the different ways in which he himself had called the Doctor over time, rather than suggesting a way *for the counsellor* to call her.

A profile of Francis as an interactional participant has emerged from the analysis of both extracts: he displays awareness of other people’s intentions as manifested in their turns at talk, can do perspective work in including relevant information that enables listeners to follow a story, and can provide information on internal states where relevant, although at times in ways that may be oblique for a neurotypical listener. However, we also observed in both examples that the way the stories were delivered made it difficult for the co-participant to engage in collaborative meaning-making or proffer validation for their content.

Turning now finally to Bruner, we will see how his theories about narrative and the self might help us tease out the psychological layers pertaining to these different types of conduct.

## Discussion

### Jerome Bruner’s Narrative Theory

When Jerome Bruner and Carol Feldman presented their theories on autism (1993) there was still a strong emphasis on higher order type of deficits at the origin of autism, and they offered hypotheses along those lines as well. However**,** I will summarise in the following Bruner‘s theories on self based on his much more substantial research on early development and family autobiographical narratives, which I believe provide a better foundation for interpreting current knowledge about autistic forms of life.

Bruner,^5^ uniquely among his contemporaries in developmental and narrative psychology, describes narrative as the exchange of intentional actions between two subjects in the present of the narrating episode. The foundations of his narrative theory are in early play between caregiver and infant, which consist in mirroring each other and engaging in repetitive sequences of simple actions. Bruner views such sequences – or ‘formats’ - as joint sense-making practices, which capture relationships in time: the ‘who-am-I-in-relation-to-the-other’ way of being in the world. Meaning emerges – in fact coincides with - the patterning of those chains of actions, i.e. of reciprocal intentionalities. Furthermore, Bruner argues, such early, embodied experiences of intentionality are already informed by culture; the world as the infant gets to know it is already a cultural world, and the inchoate self that is spun in these transactions already a cultural self. Culture, thus, in ‘Brunerian’ psychology, is a particular way of assembling actions and counter-actions, a variation in the dance of intersubjectivity which is otherwise universal.

Culture played out in interaction provides people with ‘folk psychology’ – a loose set of theories about the world and the mind - that varies not only across broad cultural communities but also across families and other groups (Bruner 1987). Folk psychology is largely implicit, but it is the psychological system ‘by which people regulate their sociality’ (Bruner and Feldman 1993: 288). Crucially, it entails repertoires of intentions. The self that is born out of this web of cultural meanings and interpersonal expectations is therefore ‘highly sensitive to bidding on the not so open market of one’s own reference group’. The self is ‘intersubjective, or distributed like knowledge’ (Bruner 1991:76). Putting all the pieces together, the self would be a position in a culturally inflected, unfolding narrative between *I* and *You*.

Bruner’s theory is consistent with developmental research showing the role of even very young infants in structuring interactions with caregivers (Tronick et al. 1977; Reddy 2008) and with language socialization research with older children showing that more expert speakers scaffold children narratives according to local theories of relevance, and append the appropriate internal responses to the child recounted events (Edwards and Middleton 1988; Ochs et al. 1989; Miller and Moore 1989).

What Bruner says about the self as a bid within the circle of one’s reference group, and its being distributed, we can see happening through story-telling in interaction in general: Conversation Analysis studies have revealed how narrators adjust stories as they go along depending on audience’ attention or appreciation, and that audiences can ultimately determine the success of narrative performances (Goodwin 1986; Ochs and Taylor 1992; Fasulo and Pontecorvo 1997), shaping over time the type of stories that will be most appropriated and shared in any given group. In this way, language precipitates entire compartments of the experience-able self (Berger and Luckmann 1966), which are, by virtue of this process, individual and cultural at the same time.

The nature of intersubjectivity that is built through co-narration goes beyond reciprocal tracking on a turn by turn basis to include alignment on the moral and generally evaluative perspective all narratives foreshadow (Ochs and Capps 2009). The self takes shape as the protagonist of certain types of stories (Bruner 1991) but needs to be continually validated through social interaction (Pasupathi and Hoyt 2009).This is why Bruner insists that, far from being backward-looking retrievals of experiential content, autobiographical narratives are forward-looking interpersonal projects, affected by interlocutors and situations in terms of the form the narrative will take, the contents that will be stressed, and the genre the narrative will be keyed into. The role of joint narrative activity in the development of cultural, interactional and autobiographical competences may help understanding the types of conduct subsumed under formulae like the paradox of the autistic self, if we consider what may be going on in the life of people with autism, especially if, like in the case of Francis, they were not diagnosed until late in life.

### Autism and Narrative Socialization

Observational research on early interaction in autism points to problems that may disrupt the natural occurring of the protonarrative interactions described by Bruner and other infant researchers. Wootton (2002) found that children with autism between 12 and 24 months produce fewer interactional initiations than typical children, and hypothesised that this may deprive them of the natural corrective responses of caregivers that contribute to communicative effectiveness. Muratori and Maestro (2007) predates these observations in their analyses of family videos of infants later diagnosed with autism: they found a lower frequency of ‘provoking’^6^ behaviours compared to typical infants of the same age, a finding that the authors discuss as affecting the ‘development of the dialogical self’. Wan et al. (2013) show that lower dyadic mutuality and infant positive affect and attention predict autism diagnosis at 3 years. Early play and proto-narratives, what Bruner called formats, are essentially embodied, relying on body tension and movements, gaze and the melody of non lexical vocalizations (Gratier 2003; Fantasia et al. 2014), all areas which develop differently in autistic children. If the continuity Bruner maintains between very early interactions, narrative co-creation and self-making is considered in all its implications, it is not far-fetched to hypothesize that the lower-order anomalies initially limiting autistic children’s initiative in interactions (Trevarthen and Delafield-Butt 2013) can generate down the line a different, less socialized, less porous type of stories in people with autism, stories with an internal, autonomous logic which is more compelling than the wish for audience validation that inhabits neurotypicals’ stories. In turn, the self that inhabits those stories may seem foreign to neurotypicals, who bring in their cultural expectations concerning the internal states that should come associated with certain events. Similarly, conventional associations between actions and intentions or emotions may also not be triggered in the interpretations of others’ conduct by people with autism, leading to the perception of a lack of a ‘theory of mind’. The finding that ‘theory of mind’ tasks are also failed by deaf children born to hearing, non signing parents (Peterson and Siegal 1999) suggests that early communication plays a significant role in developing the intuitive pre-reflexive understanding of others that can later support conventional interpretation of others’ conduct.

My hope is that the considerations above can contribute to the debate about the perceptions of the weak self and ego-centredness in autism, at least narrowing down and separating out the levels at which interpersonal misalignments and unmet expectations can occur; more importantly however I wish to reinforce the growing chorus of voices urging for a clear turn in psychological research in autism. In the last part of the discussion I will briefly point out two of the most urgent problems that should be addressed in future studies.

### Psychology and Autism Research

As mentioned earlier in the article, psychological theories on the self in autism that have been summarized with the expression ‘paradox of the autistic self’ have been criticized, on the one hand, because they are at odds with observations and accounts of people in the spectrum, and, on the other, for shortcomings in the construct of self that is adopted in these theories.

For the first part of the problem, the evidence shows it is time for autistic research to dislodge itself from its normative standpoint and go meet autism on its own ground. This would mean adopting research strategies that can open new vistas into the alternative paths through which the autistic self is created.^7^ Experimental research should find, first of all, more advantageous comparison groups than non-descript normo-gifted individuals, and secondly explore in their own right what may be distinctive systems of attention, intentional interpretations and self-other relations, rather than exposing deviations from standard neurotypical conduct. A promising avenue is the much neglected area of sensory and motor issues (Gallagher and Varga 2015), which not only have been found to interfere with the possibility of interactional engagement in early childhood (McCleery et al. 2013; Markram and Markram 2010), but also continues to pose challenges throughout the life of people in the spectrum.

Ethnographic and interactional studies represent another research avenue that can get us closer to autism in its own right. Ethnographic research has revealed forms of autistic sociality and expertise (Ochs et al. 2001; Mattingly 2017; Lawlor and Solomon 2017). Especially when using microanalytic methods for the study of spontaneous conduct, ethnographic studies have been able to unpick different layers of competencies and difficulties and the role of interlocutors in their emergence (Maynard 2005; Fasulo and Fiore 2007) and have revisited seemingly odd behavioral conducts to reveal they were adjustments to interactional demands (Tarplee and Barrow 1999; Sterponi and Fasulo 2010, Sterponi et al. 2015). Milton, a researcher in autism and in the autistic spectrum himself, advocates for ‘micro-sociological perspectives in conjunction with phenomenological and discursive methods’ (Milton 2013) and, challenges scholars to experience autistic sociality directly by engaging with people with autism:

‘I argue that some level of interactional expertise must be possible, as no autistic person is completely uncommunicative. The inter-actional expertise shown by non-autistic social researchers is, however, often clearly insufficient, […]. Gaining expertise in what it is to be autistic would take immersion in the culture and practices of autistic people […] In my view, the level of embodiment needed for interactional expertise with autistic people remains unanswered.’ (Milton 2014, 797–800)

If Milton’s proposal seems radical, it should be noted that it has solid foundations in phenomenological approaches, such ethnomethodology (Garfinkel 1967), which is the theoretical paradigm Conversation Analysis was born into, and is part of a general movement toward ‘second person perspective’ in psychological research now endorsed also in experimental cognitive science (Schilbach et al. 2013).

The second problem, about definitions of self in psychological research could take us too far, but it needs to be mentioned that many studies pointing to the pitfalls of the autistic self by implications reify simplistic views of self in typical psychological functioning. Notably, many comparative studies imply that non autistic people have immediate access to their beliefs and emotions, and that the verbal expression of the latter is the result of this direct apprehension.^8^ However, if we travel to different regions of psychological theorization, we find out that the idea of a direct access to self and internal states has been under attack for a long time. In narrative psychology the self has long been spoken of as an ‘invention’ (Eakin 1985) similar to fiction writing. Theories of autobiographical memory as providing direct access to past experiences have been debunked (see Brockmeier 2015, for a thorough review of the evidence). Recent theories of self based on both philosophical theorisation and experimental evidence propose that there is no content to the self as such, our experience of an internal self being entirely virtual and processual, the product of the way the mind works (Metzinger and Gallese 2003). Specifically, according to Metzinger and Gallese, failing to appreciate the made-up nature of self leads scientists to become ‘naïve realists’:

Frequently, the theoretical model we design about ourselves as cognitive agents is one of organisms, which, ad libitum, *direct the beam of their “epistemic flashlight” at parts of the world or their own internal lives*. […] This can lead to the kind of fallacy, which […] can also be generated in the context of representationalist theories of mind by *mistakenly transporting what [Dennett] called the “intentional stance”* (Dennett 1987) *into the system*. (Metzinger and Gallese 2003: 566 , italics added)

In other words, many models of normal psychological functioning are built on the shaky grounds of the existence of a substantial self, which is based on the phenomenal experience of having one, thus confusing explanans and explanandum. Such confusion can lead to even more misled theories when the idea of a substantial self becomes the basis for explaining deviations from the norm like that of autism.^9^

The combination of normo-centric research paradigms and unexamined assumptions about psychological processes in typical individuals (see also Gallagher 2004)– with perhaps some revelling in sensationalist language - has led to characterizations of autism as a natural experiment in human sociality, a condition that can shed light on what is quintessentially human – be it self-awareness, empathy, understanding other minds - because it creates humans who are lacking in it. This research risks tacitly perpetuating representations of autistic people as aliens (Hacking 2009), and raising barriers not only to social exchange but to good science too; Mc Donagh addresses this point in his discussion of research on empathy in autism:

Perhaps most important, it points to a disturbing movement in writings that use autism as a test case for validating the concept of empathy. This strategy succeeds only at the cost of creating *new exiles.* If empathy is seen as fundamental to humanity, those who are not particularly empathic (according to whatever definition of the term is dominant) thus acquire identities defined by this lack and pathologized by their resistance to therapy. Like the Age of Meritocratic Intelligence before it, the Age of Empathy will strive to create its *barbarians* to be placed *outside the gates*. Mc Donagh (2013 : 48, italics added)

Psychology could perhaps become a better science if it examined more carefully its tacit assumptions and reconnected to its philosophical roots in order to avoid smuggling in, as a by-product of its research paradigms, disturbing and unethical ‘images of man’ (Shotter 1975).

## Conclusions

Conversation Analysis was used in this study to examine in detail two narratives of a person with autism during counselling, selected because they could illustrate how perceptions of weak self-referentiality and of ego-centredness in autistic people could arise. The analysis revealed that, rather than from specific narrated contents, such perceptions could derive from the fact that the stories did not embed opportunities for the co-participant to intervene in the joint realization of meaning; interactional alignment was found at the level of turn-by-turn organization and informational perspective-taking, but there was no apparent need to have the recounted experience validated by the interlocutor, as is mostly the case in story-telling in both everyday and psyhcotherapeutic settings.

I argued that Bruner’s theories about early interaction and the development of self through narrative exchanges, if applied to such observations, suggest that early disruptions in basic coordination skills might reduce opportunities for training in intersubjectivity and gradually learning, from within experiences of co-narration, how to structure one’s stories in conventional narrative form, and displaying a culturally recognizable self. People with autism, with all the differences that different biographical paths entail, may thus draw on partially different resources in assembling a sense of who they are, an may develop a self more autonomous from the social environment than is generally the case for neurotypical individuals. However, this would still represent an ‘adaptive common variant pathways of human functional brain development’, as Johnson (2017:5) puts it, rather than evidence of a faulty component preventing people with autism to reach full humanity. Overall, this study attempted to show that the analysis of naturalistic data can help uncover the logic of autistic behaviour and orient autism studies toward more fruitful research approaches.

## Appendix: Transcription Symbols

: Colon(s): Extended or stretched sound.

__ Underlining: Vocalic emphasis.

(.) Brief pause, less than (0.2).

(1.2) Timed Pause: Intervals occurring within and between same or differentspeaker’s utterances in tenths of seconds.

(()) Double Parentheses: non verbal actions, contextual information.

(don’t/won’t) Single Parentheses: Transcriptionist doubt (best guess) or (guess/other guess).

. Period: Falling vocal pitch.

? Question Marks: Rising vocal pitch.

WORD Caps: Extreme loudness compared to surrounding talk.

[Brackets: beginning point at which current talk is overlapped by other talk.

*[*Bracket in italics: Simultaneous non verbal action

↓↑ Arrows: Pitch resets; marked rising and falling shifts in intonation.

= Equal Signs: Latching of contiguous utterances, with no interval or overlap.

° ° Degree Signs: A passage of talk noticeably softer than surrounding talk.

> < Less Than/Greater Than Signs: Portions of an utterance delivered at a pace noticeably quicker (> <) or slower (< >) than surrounding talk.

-Hyphens: Halting, abrupt cut off of sound or word.

.hhh: Audible inbreaths.

h h: Audible outbreaths, as for example in laughter or sighing.

wo(h)rd(h) Outhbreaths within words, as in laughter interspersed in speech.

## Footnotes

1 For an initial list see http://www.researchautism.net/published-biographies-autism. Williams (2009) and Higashida (2013) have been among the most illuminating ones for me.

2 Markram and Markram, who are proponent of the Intense World Theory (2010), have a rather stark view about the deficit in ‘self encoding’: ‘This is just another theory that is a relic of the theories of mental retardation. It is contaminated by older theories that there is a deficit in the ability of the brain to develop a theory of mind, the ability to see and respect others’ thoughts feelings and emotions. This archaic theory has also led to gross misinterpretations of the mirror neuron discoveries’ (Markram and Markram 2012).

3 All procedures performed in studies involving human participants were in accordance with the ethical standards of the University of Portsmouth – Department of Psychology Ethics Committee (Ethics approval 29/11/2012) and with the 1964 Helsinki declaration and its later amendments or comparable ethical standards.

4 The meaning of ‘white face’ was explained by Francis himself when I asked him for help with the transcription.

5 The synthesis below, where not otherwise stated, is based on the part written by Bruner in Bruner and Feldman (1993). Authorship responsibilities for the different parts of the chapter are clearly distinguished by the authors, and each section is written in the 1st person singular.

6 Muratori and Maestro (2007:97) cite Nagy and Molnár (2004) about ‘provocation’ in human behavior as an essential component of subjectivity and motivational processes.

7 In a previous study, I discuss how in case of limited reliance on in vivo interactions for the development of self, literary and historical characters can become dialogical counterparts to make sense of oneself and others (Fasulo (2014).

8 For example, a low frequency of epistemic terms (i.e. words referring to knowledge) is explained by lack of *access* to one’s own beliefs (Tager-Flusberg 1993:151); if people with autism use references to internal states, such as emotions, the way they use them is said to suggest ‘a limited understanding of what they [the emotional terms] *really* stand for’ (Colle et al. 2008: 38); the scarcity of words referring to internal states in autobiographical narratives of 6–14 years are interpreted as ‘consistent with the possibility that children with AD *lack awareness of their own reactions* to personal experiences’ (Brown et al. 2012:44) (all italics added).

9 See Scholiers (2019) for a theory of the self in autism that attempts to integrate this model of self and Makram and Makram’s Intense World Theory.
